# Nasal dominance potentiates intranasal oxytocin’s anxiolytic effects

**DOI:** 10.1038/s41598-025-95148-x

**Published:** 2025-05-27

**Authors:** Nikita Catalina Julius, Dasha Nicholls, Joseph Nowell, Victoria Burmester

**Affiliations:** Division of Psychiatry, Department of Brain Sciences, Imperial College London, Du Cane Road, W12 0NN London, UK

**Keywords:** Emotion, Social behaviour, Stress and resilience, Human behaviour

## Abstract

The nasal cycle is a phenomenon whereby alterations in airflow alternate between left and right nostrils. During a nostril’s decongested – or dominant – state, the contralateral nostril is congested, or non-dominant. Intranasal oxytocin may elicit anxiolytic effects. To date, no study has investigated whether there is an optimal nasal pathway for oxytocin’s effects. Forty-four female adolescents aged 16 to 17 years were included in this exploratory study investigating effects of nasal dominance on intranasal oxytocin delivery. We show that intranasal oxytocin significantly reduces stress relative to placebo (*p* = 0.012, ηp^2^ = 0.145) and greater stress reduction occurs when oxytocin is delivered to the dominant, rather than non-dominant, nostril (*p* = 0.034, ηp^2^ = 0.113). We postulate that oxytocin administration may reduce stress and be most effective in the context of anxiolysis when administered to the dominant nostril. Further research investigating whether other intranasal psychotropic drugs have nostril-specific effects might benefit clinical practice.

## Introduction

The nasal cycle is characterised by rhythmic alterations in airflow between the left and right nostrils, where congestion in one nostril is cyclically accompanied by decongestion in the other nostril^1^. This phenomenon is seen in approximately 70–80% of healthy adults, switching states approximately every two hours in wake and 4.5 h in sleep^2^. Although significant variability in cycle length has been reported^3–5^, the cycle frequency can be indirectly affected by factors such as body mass index (BMI)^6^. In the decongested nostril, referred to as the dominant nostril, there is increased airflow. In the contralateral nostril, also known as the congested or non-dominant nostril, airflow is decreased, marking the ‘resting phase’ of the cycle^7^. It is currently thought that dominance is related to vasoconstriction in the decongested nasal cavity, while non-dominance is associated with vasodilation in the congested nasal cavity^8^.

Oxytocin is a nonapeptide, primarily synthesised in the hypothalamic supraoptic and paraventricular nuclei and released by exocytosis into the central nervous system^9^. Oxytocin receptors are the only described receptor expressed in neural tissue and other parts of the body in species-dependent patterns^10^. Owing to its molecular mass of 1007 Daltons^11^, oxytocin was previously considered unable to penetrate the blood-brain barrier (BBB). However, recent findings have demonstrated that oxytocin may bind to the receptor for advanced glycation end-products (RAGE), facilitating its transport across the BBB^12,13^. Oxytocin has a wide role in the stress response, acting as an anxiolytic agent via the hypothalamic-pituitary-adrenal axis^14^. Pierrehumbert et al. induced laboratory stress in humans using the Trier Social Stress Test to investigate the presence of an oxytocin response and found a negative correlation between oxytocin and cortisol, suggesting an interaction between cortisol release and oxytocin^15^.

Intranasal oxytocin is widely used in psychological and psychiatric studies. The mechanism by which exogenously administered oxytocin elicits its effects on brain and behaviour is still unclear, in part due to the absence of a positron emission tomography radiotracer approved for human use. There are three main theories, although not necessarily competing, explaining how oxytocin is absorbed through the nostril to effect central change. The direct route theory assumes that oxytocin circumvents the BBB through an extracellular pathway involving the olfactory and trigeminal nerves and perineural clefts in the nasal epithelium^16^. Once intranasal oxytocin reaches the olfactory and respiratory epithelia, it is transported via the ensheathed channels surrounding the olfactory and trigeminal nerve fibres^17^. The olfactory nerve fibres then pass through clefts in the cribriform plate, leading to the olfactory bulbs. The BBB theory posits that oxytocin may be delivered to the brain by crossing the BBB in small but biologically relevant quantities^18^. Recent findings substantiate oxytocin’s transport across the BBB following exogenous administration via RAGE-mediated transport^13^. Thirdly, oxytocin administered intranasally may have the potential to influence afferent feedback to the brain from peripheral systems, specifically from organs abundant in oxytocin receptors, thereby influencing central oxytocin function^19^.

Although the transport of oxytocin from nose-to-brain is not fully articulated, understanding the influence of nasal dominance on oxytocin absorption may shed light on oxytocin’s pathway to the brain. We hypothesised that there would be a difference in stress scores following administration of a stressor depending on whether intranasal oxytocin was delivered via the dominant or non-dominant nostril.

## Results

### Participant characteristics

A convenience sample of 44 adolescent females aged 16–17 years (Table 1) with BMI from 15.61 to 29.75 kg/m^2^ [*M (SD)* = 21.19 (3.91) kg/m^2^] were included in this study.

**Table 1:** Participant sociodemographics.

	Total, *n* = 44
Ethnicity, n (%)	
White	16 (36.4)
Black	11 (25)
Asian	9 (20.5)
Mixed	6 (13.6)
Other	2 (4.5)
Social support relationship, n (%)	
Friend	29 (65.9)
Parent/guardian	8 (18.2)
Sibling	4 (9.1)
Romantic partner	2 (4.5)
Other	1 (2.3)
Parental education, n (%)	
At least one with university degree	25 (56.8)
No university degree	16 (36.4)
Unsure	3 (6.8)

The dominant group consisted of 23 participants, while the non-dominant group consisted of 19 participants. One participant was assigned to the non-dominant group but without an obvious state determined on the second visit; this visit was counted as missing data. One participant was assigned different states on each visit due to a procedural error. Comparisons between the groups can be seen in Table 2.

**Table 2:** Group comparisons.

Comparison	Mean (SD)	t	df	*p*-val
ED status		0.139	41	0.890
Dom	0.521 (0.511)			
Non	0.500 (0.513)			
BMI		1.883	40	0.067
Dom	20.245 (3.625)			
Non	22.478 (4.025)			
RCADS-SP		0.404	41	0.688
Dom	16.565 (5.655)			
Non	17.250 (5.408)			
Baseline stress		0.226	41	0.822
Dom	2.957 (2.246)			
Non	2.800 (2.285)			
Contraceptive use		0.143	41	0.887
Dom	0.090 (0.288)			
Non	0.100 (0.308)			
Use of vapes or cigarettes		0.604	41	0.549
Dom	1.870 (0.344)			
Non	1.800 (0.410)			

### Shortened Sing-A-Song Stress test as a stressor

T tests were employed to investigate the adequacy of the shortened Sing-a-Song stress test (SSSTs) as a stressor. SSSTs significantly increased self-reported stress when compared to baseline self-reported stress in both placebo, t(43) = 3.087, *p* = 0.002, d = 0.468, and oxytocin conditions, t(43) = 4.473, *p* < 0.001, d = 0.674.

### Effects of oxytocin on stress

**Fig. 1:**
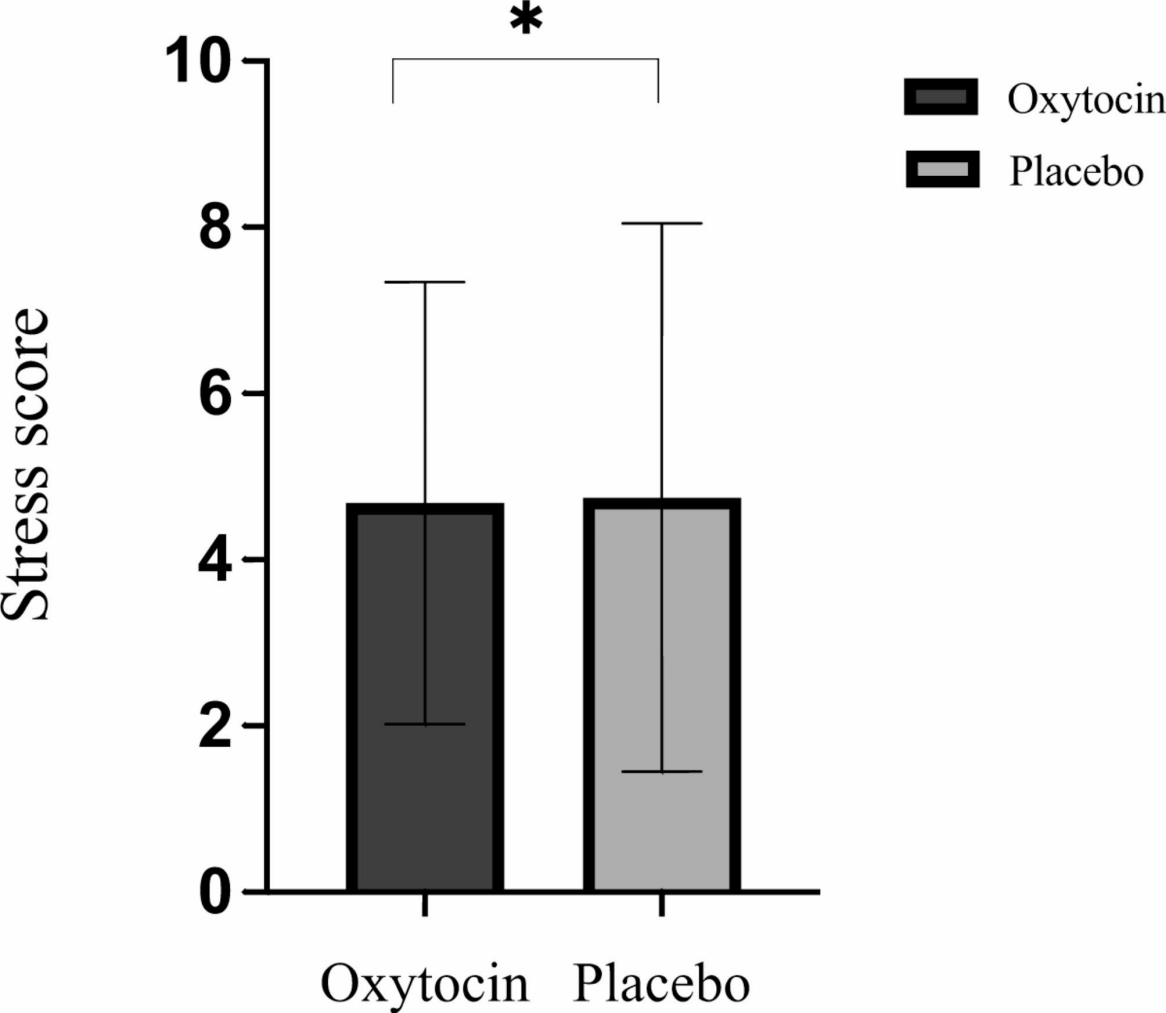
Bar chart representing stress scores in the oxytocin and placebo conditions (± SD) after the Sing-A-Song Stress Test when adjusting for baseline stress, eating disorder status, and RCADS-SP, *p < 0.05.

A repeated-measures analysis of covariance (RM-ANCOVA) evaluated the effects of oxytocin on stress scores after the SSSTs between the oxytocin and placebo conditions. RCADS social phobia (RCADS-SP) scores and eating disorder (ED) status were used as covariates in the model. Results indicated that that oxytocin significantly reduced stress [*M (SD)* = 4.68 (2.66)] relative to placebo [*M (SD)* = 4.75 (3.30)], with a large effect: F(1,41) = [6.951], *p* = 0.012, ηp^2^ = 0.145 (Fig. 1). Additionally, a significant interaction was found between oxytocin’s effects and RCADS-SP scores, with medium to large effect size: F(1,41) = [6.632], *p* = 0.014, ηp^2^ = 0.139.

Sub-group analyses were conducted to further explore the interaction between oxytocin’s effects and RCADS-SP scores. Participants were categorised into two groups based on RCADS-SP scores: those with ‘elevated’ social phobia scores of 20 and above (*n* = 15) and those with ‘normal’ scores of zero to 19 (*n* = 29), as per Child Outcomes Research Consortium interpretation guidelines^20^. For the elevated RCADS-SP group, an RM-ANCOVA controlling for ED status revealed a significant reduction in stress scores in the oxytocin condition [*M (SD)* = 5.00 (2.45)] compared to placebo [*M (SD)* = 6.20 (2.70)], with a large effect: F(1,13) = [8.720], *p* = 0.011, ηp^2^ = 0.401. In contrast, no difference in stress scores between the oxytocin [*M (SD)* = 4.52 (2.79)] and placebo [*M (SD)* = 4.00 (3.37)] conditions was observed for the normal RCADS-SP group: F(1,27) = [2.938], *p* = 0.098, ηp^2^ = 0.098.

### Nostril dominance

**Fig. 2:**
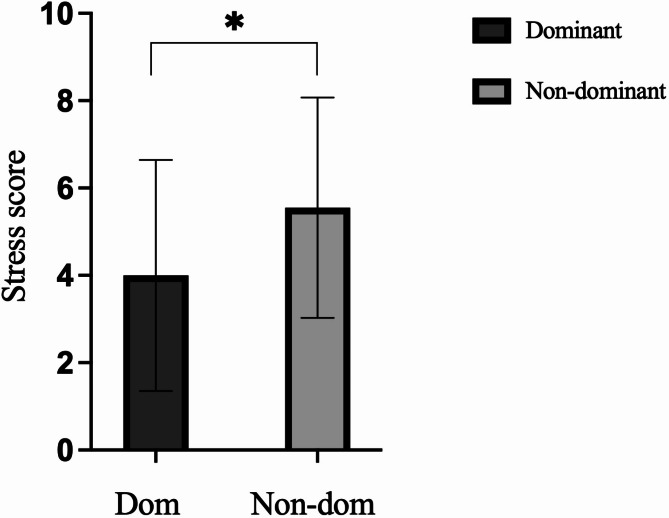
Bar chart representing stress scores by nostril (± SD) after the Sing-A-Song Stress Test when adjusting for baseline stress, eating disorder status, and RCADS-SP, *p < 0.05.

An ANCOVA evaluated the effects of nostril dominance on stress scores after the SSSTs in the oxytocin condition with baseline stress scores, RCADS-SP scores, and ED status as covariates. Results indicated that oxytocin elicited a significant difference in stress score by nostril after the SSSTs with a medium-large effect: F(1,42) = [4.831], *p* = 0.034, ηp^2^ = 0.113 (Fig. 2.). Post hoc testing using Fisher’s Least Significant Difference (LSD) indicated that the stress score was significantly lower when oxytocin was delivered intranasally to the dominant nostril [*M (SD)* = 4.00 (2.65)] compared with the non-dominant nostril [*M (SD)* = 5.55 (2.52)], *p* = 0.034.

**Fig. 3:**
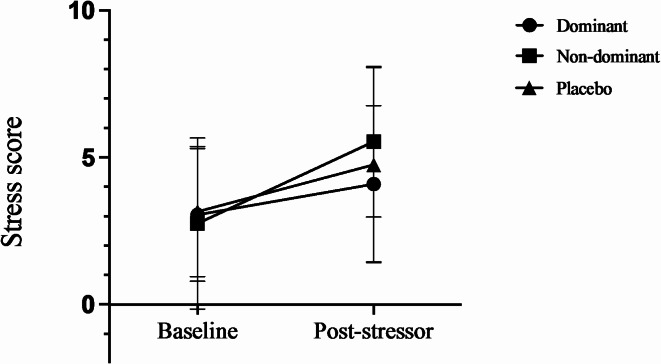
Line graph representing baseline and post-Sing-A-Song Stress Test stress scores (± SD) in the placebo (*n* = 44), dominant (*n* = 22), and non-dominant conditions (*n* = 20).

Only baseline stress served as a significant covariate in the model: F(1,42) [6.442], *p* = 0.015, ηp^2^ = 0.145. Stress scores for the dominant and non-dominant oxytocin groups, as well as the placebo groups, relative to baseline stress scores, can be seen in Fig. 3.

### Nostril dominance in placebo condition

An ANCOVA evaluated the effects of nostril dominance on stress scores after the SSSTs in the placebo condition with baseline stress scores and RCADS-SP scores as covariates. The results indicated that there was no significant difference in stress score by nostril: F(1,42) = [1.818], *p* = 0.186, ηp^2^ = 0.046.

## Discussion

To the best of our knowledge, this study is the first to explore whether intranasal oxytocin’s anxiolytic effects are affected by nostril dominance. Administration of 24 international units (IU) of intranasal oxytocin significantly reduced stress when compared with placebo. Intranasal oxytocin showed a significant increase in effectiveness in reducing self-reported stress when applied to the dominant nostril compared to the non-dominant nostril.

Following induction of stress using the modified SSSTs, oxytocin significantly decreased self-reported stress relative to placebo, indicating its potential role in mitigating social-evaluative stress. This effect was seen when controlling for social phobia (SP) symptomatology and ED status. This finding aligns with literature implicating oxytocin as an anxiolytic agent. Without accounting for SP symptoms, there was no significant reduction in social-evaluative stress, underscoring oxytocin’s interindividual-dependent properties, specifically social anxiety symptomatology^21^.

The significant interaction between oxytocin’s effects and RCADS-SP scores suggests that oxytocin’s anxiolytic properties may be influenced by trait SP. Specifically, participants with elevated SP scores experienced a significant reduction in self-reported stress following oxytocin administration, while those with normal scores did not show this effect. This finding also supports the social salience hypothesis of oxytocin that proposes the salience effect depends on baseline individual differences, such as personality traits and degree of psychopathology^21^. It is important to note that our elevated SP group was small (*n* = 15), which may account for the subtle overall reduction in stress scores between the oxytocin and placebo conditions. This limitation warrants further research with larger sample sizes. These results highlight the possibility that oxytocin’s protective effects against social-evaluative stress may be more pronounced in individuals with higher SP and less effective in those without such traits, underscoring the need to consider these individual differences for therapeutic purposes.

The dominant nostril elicited significantly lower stress scores compared to the non-dominant nostril in the oxytocin condition. This result suggests that, for the purpose of anxiolysis, oxytocin might be more efficacious when administered to the dominant than the non-dominant pathway. This is an important finding as it indicates that there may be a preferred nostril for optimal oxytocin administration in the context of stress reduction. In our sample, the significant nostril-specific effect of oxytocin might be attributed to several factors. We implemented a procedure to identify the dominant or non-dominant nostril for oxytocin administration and identified the presence of definitive nostril states in 98% of our sample. This procedure involved clearing the nasal passage by exhaling into a tissue and obtaining a self-report of the dominant nostril, which was objectively confirmed using the Glatzel mirror test^22^, although it is important to note that these methods may not rule out any nostril abnormalities. Additionally, participants received a demonstration of the self-administration procedure by a researcher and were provided with printed instructions. We excluded participants with hay fever and nasal congestion to maintain consistency in the sample. Our sample was homogenous, comprising only females aged 16–17. Participants with a BMI > 30 kg/m^2^ were excluded from all analyses to mitigate any influence of BMI-related effects, such as increased airflow; there is no evidence that BMI affects the nasal cycle or inhibits dose-responsivity^23–25^.

We postulate that the observed differences in stress scores between nostrils in the drug condition might be attributed to the effects of oxytocin as we found no nostril-specific response in the placebo condition. The interplay between oxytocin’s effects, placebo responses, and nostril-specific variations in stress scores present a complex interaction, warranting further research.

This exploratory work aligns with literature suggesting that the olfactory and trigeminal nerves might function as the optimal route for oxytocin delivery to the brain within the window of approximately 20–90 min^26^. In this timeframe, the dominant nostril, with constricted blood vessels, might provide a more direct pathway to the brain, while the non-dominant nostril, with dilated blood vessels, could lead to greater absorption into the peripheral system before being transported to the brain. Oxytocin’s delivery to the brain via these pathways is also influenced by its uptake speed. The significantly greater reduction in stress scores we observed when oxytocin was administered to the dominant nostril supports existing literature that suggest the olfactory and trigeminal nerve routes may play a more substantial role in oxytocin’s nose-to-brain delivery than the peripheral route during the putative peak effects of oxytocin^27,28^.However, we acknowledge the possibility that the peripheral pathway might also contribute to its effects differentially. Further investigation, perhaps considering dose-response, uptake speed, and timeframe, is needed. In addition to the conventional nasal spray, an alternative method of administering oxytocin via the nasal passage involves the use of a nebuliser. A nebuliser is purported to improve oxytocin deposition in specific nasal regions directly implicated in transportation to the brain^29^. Employing this method of delivery could shed more light on the mechanisms involved in nose-to-brain transport, offering more evidence for the nostril-specific effect.

This study has its limitations. Although a significant increase in stress was observed from baseline readings to post-stressor in both the oxytocin and placebo conditions, we cannot exclude the possibility that preceding tasks contributed to changes in subjective stress. Additionally, our stressor primarily applies to social-evaluative stress and may not be applicable to general stress without further investigation. Moreover, the Glatzel mirror test cannot definitively determine nostril dominance/non-dominance; abnormalities such as nasal septal deviation that may affect the determination of nostril states require further examination through imaging techniques and nasal endoscopic assessments. Additionally, while our use of self-reported Visual Analogue Scale (VAS) stress scores yielded significant results as an outcome measure, future studies could employ objective measures, such as skin conductance, heart rate variability, salivary cortisol measures, or neuroimaging techniques to increase sensitivity and minimise subjectivity in capturing stress responses. Data on days since the last menstrual period and hormonal contraceptive use were collected; however, an accurate phase could not be calculated due to the variable length of menstrual cycles in our sample, which included individuals with EDs^30,31^, and no data on type of contraceptive (progesterone, oestrogen and progesterone, the coil) collected.

Further, there are various administration positions for intranasal drug delivery, each influencing deposition differently^32^. While we opted for the basic head-tilt position as it required less movement, which could potentially serve to reduce external stressors, future research should explore alternative positions to enhance the deposition of the active drug in optimum regions of the nasal cavity. The Mygind and ‘praying-to-Mecca’ positions are suggested to deposit drugs closer to the olfactory nerves^32^ and thus may offer improved efficacy for nose-to-brain delivery.

This preliminary work should be replicated in a larger, more diverse sample, including males^33,34^ and exploring different doses of oxytocin, timeframes, and- other intranasal psychotropic drugs, such as esketamine^35^. The potential clinical implications could be substantial. If the nasal cycle state indeed influences responses to intranasal delivery, determining the optimal nostril for administering drugs targeting the brain could have significant implications for effectiveness and efficiency. This theory could be extended to individuals with a clear nasal cycle, suggesting that adjusting drug volume based on the most effective nostril could have pharmaceutical implications. Additionally, future research could investigate intranasal drugs targeting sites aligned with the peripheral system and whether there is an optimal nostril for their effects. Intranasal oxytocin is also employed in other contexts, such as stimulating the milk-ejection reflex in breastfeeding mothers. Our findings suggest that administering oxytocin to the dominant nostril may expedite lactation and potentially lower the required dosage.

## Conclusion

This is the first study of its kind to examine nostril-specific effects of oxytocin. We show that 24 IU of intranasal oxytocin significantly reduces social-evaluative stress and is significantly more effective in this stress reduction when administered to the dominant nostril compared to the non-dominant nostril. Future work should aim to corroborate this in a larger sample, with objective methodologies, and utilise other psychotropic drugs.

## Methods

### Design

This exploratory study was part of a larger placebo-controlled, double-blinded, randomised crossover trial investigating a range of social functions in adolescent females with EDs. Participants were tested twice, approximately seven days apart [*M (SD)* = 7.95 (6.24)].

### Participants

Forty-eight adolescent females aged 16 and 17 years were recruited via social media campaigns. Four participants were excluded from the final analyses for this study due to their BMI exceeding 30 kg/m^26^.

To confirm eligibility, participants completed an online Qualtrics screening survey. The inclusion criteria required that participants were females aged 16 to 17 years, able to travel to the Children’s Clinical Research Facility at St. Mary’s Hospital, London. Exclusion criteria included insufficient English language skills and uncorrected hearing or sight impairment; regular or accomplished solo singers; pregnancy or breastfeeding; or recent experience of a significant life event. Participants entered their weights and heights, and it was noted whether they took any contraceptives due to the potential influence of menstrual cycle on endogenous oxytocin concentrations.

### Materials

Intranasal sprays containing 24IU^23–25^ of Syntocinon^®^ or vehicle were supplied by Victoria Apotheke, Switzerland and over-the-counter urine pregnancy tests were provided.

### Self-report questionnaires

A bespoke questionnaire via Qualtrics collected information on participant gender, age, ethnicity, parental education level, type of social support, date since last period, time since most recent meal, caffeine consumption, hours slept the previous night, and handedness.

A digital VAS scale with a sliding marker from 0 to 10 anchored by “not at all” and “as much as I can imagine” asked how stressed the participant felt at baseline and post stressor. Other measures included in the parent study were not analysed here.

The 9-item Revised Children’s Anxiety and Depression scale – Social Phobia subscale^36^ determined SP symptomatology. For each statement, participants selected one of four responses: ‘Never’, ‘Sometimes’, ‘Often’, and ‘Always’. Possible scores for the SP subscale range from 0 to 27, with scores between 0 and 15 considered a ‘normal range’ for this population. Other measures included in the parent study were not analysed here.

### Shortened ‘Sing-a-Song’ Stress test

Singing to an audience is a reliable method for inducing social-evaluative threat in participants. A shortened SSSTs that required a 50 s singing performance, was used^37^. After choosing a song, participants stood and performed it in front of the research team and the person accompanying them. As part of the sham story, and to increase stress, the researchers were seen to be scoring the participant’s performance. Nursery rhymes, humming and children’s songs were not allowed as these were considered less stressful to perform.

### Procedures

The study was sponsored by Imperial College London and approvals were obtained from the Leicestershire South Research Ethics Committee (reference: 22/EM/0044; IRAS project ID: 297695). All methods were carried out in accordance with relevant guidelines/regulations. Testing was carried out in the Clinical Research Facility at St Mary’s Hospital, London. An independent statistician carried out randomisation.

Each visit lasted approximately one hour, and participants were instructed to bring a social support who remained present throughout the visit. This recommendation is based on evidence suggesting that the presence of a social support figure may enhance oxytocin’s effects^38^. Participants and their social support were compensated through a £50 and £20 online voucher, respectively, upon completion of both visits.

Participants were told that the study was investigating whether oxytocin, an agent that may reduce anxiety and increase sociability, impacts social behaviour in adolescent females with and without ED. To minimise biases, both researchers and participants and their social supports were blinded to whether subjects met the criteria for an ED.

At the start of the first visit, participants verbally confirmed eligibility and provided informed consent electronically. Experimenters noted whether they had consumed alcohol in the last 12 h, smoked or vaped, or had any conditions causing nasal congestion on the day. After participants cleared their nasal passages using a tissue, with a closed mouth, they forcefully exhaled through each nostril while keeping the other nostril closed, and self-reported which nostril felt more, and less, blocked. The decision on left or right nasal dominance was then confirmed by researchers, using the Glatzel Mirror test^22^. Participants and social supports were not informed about the purpose of this test or the parent study aims. After reading printed instructions, watching a demonstration on how to operate the bottle, and practising operating an identical device filled with water, participants proceeded to self-administer either placebo or 24IU oxytocin by closing one nostril with their finger, tilting their head^39^ and inhaling once, with a 30 s interval to allow for maximum absorption in either the dominant or non-dominant nostril. Researchers stayed to witness the self-administration. The selection of the nostril was pre-determined using a pseudo-randomised alternating design.

**Fig. 4:**
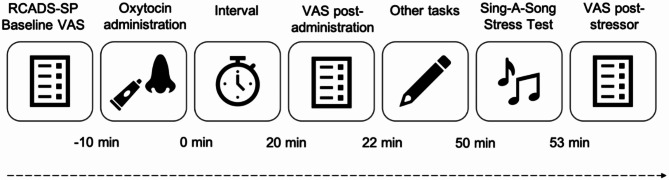
Approximate experimental timeline.

Oxytocin’s peak effects typically manifest between 20 and 90 min following intranasal administration^26^. Following a series of additional tasks detailed in the parent study, participants underwent the SSSTs, approximately 50 min post oxytocin administration. All tasks were administered in the same order for each task and task order was not counterbalanced to maximise equality of central oxytocin across tasks^40–42^. Stress VAS was administered before concluding the testing session, see Fig. 4 for the timeline of events.

### Data

Data were analysed using Statistical Package for the Social Sciences (SPSS) software version 29. A *p-value* < 0.05 was considered statistically significant. Two-tailed t-tests were employed to investigate whether there were differences in stress scores between baseline and post-stressor readings in both the oxytocin and placebo conditions, two-tailed t-tests were also employed to rule out any group differences between the dominant and non-dominant groups for ED status, BMI, RCADS-SP, baseline stress, contraceptive use, and use of vapes or cigarettes. RM-ANCOVA were performed for stress scores in the oxytocin and placebo conditions with RCADS-SP, and ED status as covariates. ANCOVAs was performed for stress scores within the oxytocin and placebo conditions, with nostril dominance as the independent factor and baseline stress score, RCADS-SP, and ED status as covariates.

## Data Availability

All data are available from the authors upon reasonable request from the corresponding author.
